# Higher Plasma Saturated and Omega-6 Fatty Acids Associated with Cholesterol Homeostasis and Gut Microbiota in Pediatric Type 2 Diabetes and Metabolic Syndrome

**DOI:** 10.34133/csbj.0194

**Published:** 2026-08-10

**Authors:** Shirley Mora-Godínez, Luis M. Marín-Obispo, Anael Mellado-Negrete, Ana L. de la Garza, Oscar Tamez-Rivera, Mariana Navarro-Guerra, Benjamín Telles-Ramírez, Emmanuel Martínez-Ledesma, Erika Castaño-Moreno, Carolina Senés-Guerrero, Dariana Rodríguez-Sánchez, Gerardo García-Rivas, Elena C. Castillo, Nora A. Rodríguez-Gutiérrez, Leticia Elizondo-Montemayor, Carmen Hernández-Brenes

**Affiliations:** ^1^ Institute for Obesity Research, Tecnologico de Monterrey, Monterrey 64849, NL, Mexico.; ^2^ Escuela de Ingeniería y Ciencias, Tecnologico de Monterrey, Monterrey 64849, NL, Mexico.; ^3^ Escuela de Medicina y Ciencias de la Salud, Tecnologico de Monterrey, Monterrey 64710, NL, Mexico.

## Abstract

Type 2 diabetes mellitus (T2DM) and metabolic syndrome (MetS) in children and adolescents are characterized by altered lipid metabolism and gut microbiota. Prior untargeted (nonquantitative) lipidomics of the present T2DM and MetS pediatric cohort revealed alterations in plasma lipids. Fatty acids (FAs) bonded to plasma phospholipids (PLs) and cholesterol esters (CEs) reflect endogenous metabolism and exogenous sources. The present study focused on targeted quantification of FAs esterified to plasma PLs and CEs in children and adolescents with T2DM and MetS and healthy controls (*n* = 60, ages 7 to 17). Regression models and Spearman correlations assessed associations of esterified FA with the disease, metabolic risk factors, pro-inflammatory cytokines, and gut microbiota composition. T2DM and MetS groups featured higher concentrations of saturated FAs esterified to PLs (C17:0) and CEs (C10:0, C12:0, and C24:0) than healthy controls. Also, both groups had higher omega-6 FA levels, including dihomo-γ-linolenic acid (C20:3n-6) in both plasma fractions (PLs and CEs), C22:5n-6 in PLs, and C18:2n-6 and C20:4n-6 in CEs. These FAs were inversely correlated with high-density lipoprotein cholesterol and directly correlated with obesity, triglycerides, and insulin resistance. Also, MetS had a high CE-omega-6/omega-3 ratio. Gut microbial taxa associated with T2DM and MetS after Tanner, sex, and body mass index percentile adjustment were *Agathobacter*, *Dorea*, *Fusicatenibacter*, and *Gemmiger*, with higher abundances than those in healthy controls, and the genus *Faecalimonas* at lower abundances. This work contributes to the current knowledge of lipid metabolism and the role of gut microbiota in T2DM and MetS in children and adolescents.

## Introduction

The incidence of type 2 diabetes mellitus (T2DM) and metabolic syndrome (MetS) has increased in children and adolescents [1,2]. Pediatric obesity is the main trigger of these comorbidities [3–5], with a global prevalence of 160 million in the pediatric population (aged 5 to 19 years) in 2022 [6]. Obesity and related comorbidities are multifactorial conditions influenced by genetic predisposition, diet, and physical activity, among other factors [4,7]. T2DM and MetS are characterized by insulin resistance and low-grade chronic inflammation [8,9]. Although they vary, in most clinical studies, the diagnostic criteria for MetS include abdominal obesity, hypertension, hypertriglyceridemia, and low levels of high-density lipoprotein cholesterol (HDL-c) [2,10]. In contrast, the diagnosis of T2DM depends on elevated plasma glucose levels [11]. Because of limited information on MetS and T2DM in children and adolescents, there is a need for further scientific research to better understand metabolic alterations and their diagnosis in this population [4].

Altered circulating lipid levels have been associated with obesity and related comorbidities in children [12–14] and adults [15–18]. Specifically, phospholipids (PLs) have been reported to be among the lipid families most frequently altered. In adults, higher levels of phosphatidylcholines and phosphatidylethanolamines and lower levels of plasmalogens and lysophospholipids (LysoPLs) were associated with T2DM risk [19,20]. In children with obesity, higher concentrations of phosphatidylethanolamines and phosphatidylinositols and lower LysoPLs have been directly associated with insulin resistance and cardiometabolic risk factors [12]. Cholesterol ester (CE) metabolism has also been found to play an important role in obesity, T2DM, and MetS [21,22]. In human metabolism, CEs are synthesized by the transfer of the sn-2 position fatty acid (FA) from a PL to free cholesterol on the surface of HDL-c by the enzyme lecithin:cholesterol acyl transferase (LCAT) [21,22]. Therefore, LCAT activity contributes to reverse cholesterol transport [21–23]. Lower LCAT activity has been reported in adult patients with MetS [21] and in children and adolescents with abdominal obesity [22].

Characteristic differential FAs esterified to complex lipids have been reported in obesity and T2DM [12,19,24]. In adults at risk for T2DM, profiles of PL-esterified FAs revealed increased linoleic acid (LA; C18:2n-6) and decreased eicosapentaenoic acid (EPA; C20:5n-3) [25]. Also, in adults, T2DM risk has been directly associated with shorter-chain CEs (14 carbons) [19] and lower levels of longer-chain FAs in CEs (18 to 20 carbons with 1 to 5 unsaturations) [20]. In children with obesity and MetS, higher levels of PL-FAs, palmitoleic acid (C16:1n-7), and dihomo-γ-linolenic acid (DGLA; C20:3n-6) have been observed [26]. The plasma levels of esterified FAs are influenced by a combination of endogenous metabolism and dietary intake [25,27].

Notably, growing evidence suggests that the gut microbiota influences host lipid metabolism, highlighting gut dysbiosis as a potential contributor to obesity-related comorbidities and lipid metabolism impairment [28–30]. Previous studies in the present cohort reported alterations in gut microbiota composition in children with T2DM and MetS [31,32]. Also, prior nonquantitative untargeted lipidomic analysis of these pediatric subjects revealed alterations in lipid metabolism, including PLs and CEs [24]. Therefore, the present work focused on targeted characterization and quantification of FAs esterified to plasma PLs and CEs in children and adolescents with T2DM and MetS and on determining their associations with metabolic risk factors, pro-inflammatory cytokines, and gut microbiota composition.

## Materials and Methods

### Study design and population

**Fig. 1.**
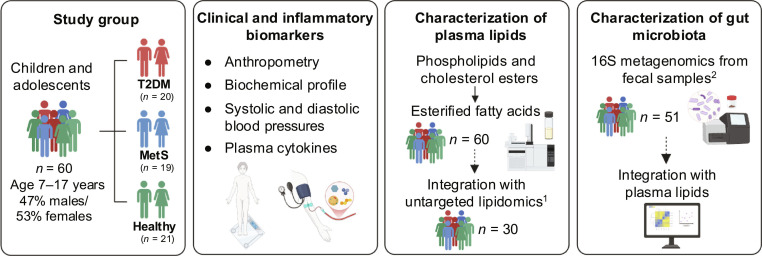
Study design for analyses of plasma fatty acids (FAs) esterified to phospholipids (PLs) and cholesterol esters (CEs) and gut microbiota composition in children and adolescents with type 2 diabetes mellitus (T2DM) and metabolic syndrome (MetS) and in a healthy group. ^1^Data from a previous study with a subset of the study group [24]. ^2^Data obtained from National Center for Biotechnology Information (NCBI) BioProject no. PRJNA819279. All data from the present and previous studies were generated from samples collected at the same time.

A cross-sectional comparative study was conducted on 60 pediatric subjects aged 7 to 17 years from a convenience sampling database at the Hospital Regional Materno Infantil de Alta Especialidad, Nuevo León, Mexico. Subjects were classified into 3 groups: T2DM (*n* = 20), MetS (*n* = 19), and a healthy control group (*n* = 21) (Fig. 1). Diagnoses of MetS and T2DM were established according to the criteria of Cook *et al.* [10] and the American Diabetes Association [11], respectively, and confirmed by a pediatrician. Subjects in the control group presented normal values for anthropometric and biochemical parameters for their age and sex. Exclusion criteria included other cardiovascular, metabolic, or pro-inflammatory diseases; abnormalities on blood samples; and use of antibiotics or probiotics in the last 3 months. The study was conducted in adherence with Good Clinical Practice guidelines. The study protocol was approved by the Ethics and Research Committee of the School of Medicine at Tecnologico de Monterrey (AIEMPPDM2SM), the Bioethics National Commission (CONBIOETICA-19-CEI-011-20161017), and the Ethics Committee of the Mexican Secretariat of Health (13 CI19039138). Parents and/or legal guardians provided written informed consent in accordance with the Declaration of Helsinki.

### Anthropometry and blood pressure measurements

Anthropometric parameters were measured using standardized protocols from the National Health and Nutrition Examination Survey [33]. Weight and height were measured using the TANITA BF-689 device (TANITA Corporation of America Inc., USA) and the SECA 217 stadiometer (SECA Mexico, Mexico), respectively. Waist circumference (WC) and hip circumference were measured using a SECA fiberglass tape. Body mass index (BMI) was calculated using the Quetelet formula, and the BMI percentile for age and sex was determined using the Centers for Disease Control and Prevention’s (CDC’s) BMI Percentile Calculator for Child and Teen [34]. The WC percentile was assigned using the CDC table for age and sex [35]. Systolic blood pressure (SBP) and diastolic blood pressure (DBP) were measured with a pediatric mercury sphygmomanometer. SBP and DBP percentiles for sex, age, and height were obtained according to The Fourth Report on the Diagnosis, Evaluation, and Treatment of High Blood Pressure in Children and Adolescents [36]. Pubertal status was assessed using the Tanner image-based self-assessment method according to Tanner stage criteria [37,38].

### Biochemical profile and cytokine analyses

Blood samples were obtained by venipuncture after a 12-h overnight fast. Serum and plasma were collected by centrifugation and stored at −80 °C. Serum fasting glucose (Glc) was measured using the Glucose 3L82 reagent kit (304772/R02, Denka Seiken Co. Ltd., Japan) and insulin using the ARCHITECT Insulin Reagent kit 8K41-27 (G6-2892/R03, Abbott Laboratories Diagnostic Division, USA) in an Architect cSystems chemistry analyzer. The homeostatic model assessment of insulin resistance (HOMA-IR) was calculated using the Matthews formula [fasting Glc × fasting insulin/405]. Total cholesterol (TC), triglycerides (TGs), and HDL-c were quantified using the Cholesterol Reagent kit 7D62 (304796/R02), the Triglyceride 7D74-20 assay (30-3140/R3), and the Ultra HDL 3K33-21 assay (306571/R03), respectively, from Abbott Laboratories Diagnostic Division, USA, in Architect cSystems. Low-density lipoprotein cholesterol (LDL-c) was calculated using Friedewald’s formula [TC − HDL − (TG/5)]. Plasma cytokine concentrations were determined using the LEGENDplex Human Inflammation 13-plex Panel (BioLegend, USA) on a FACS-Canto II flow cytometer (Becton Dickinson, USA).

### Quantification of esterified FAs in plasma PLs and CEs

Lipidic extracts were obtained from 200 μl of plasma samples using the Folch method [39]. Lipids were fractionated into lipid classes using a solid-phase extraction aminopropyl column (500 mg, 3 ml, Bond Elut NH2, Agilent Technologies Inc., USA) following the protocol described by Agren *et al.* [40]. Cholesteryl nonadecanoate (400 ppm; Nu-Chek Prep Inc., USA) and diheptadecanoyl phosphorylcholine (400 ppm; Abcam, UK) were used as internal extraction standards. PL and CE fractions were transmethylated to produce fatty acid methyl esters (FAMEs) as previously reported by Castillo *et al.* [41]. Cholesteryl undecanoate (600 ppm; Nu-Chek Prep Inc., USA) was added as an internal standard for quantification. FAMEs from PL and CE fractions were analyzed using an Agilent 6850A gas chromatograph equipped with a flame ionization detector (Agilent Technologies Inc., USA) and an SP-2380 fused silica capillary column (100 m × 0.25 mm i.d., 0.2-μm film thickness; Supelco, USA). The injector and flame ionization detector temperatures were set at 260 and 300 °C, respectively. A FAME standard mixture (GLC 566, Nu-Chek Prep Inc., USA) was used for identification and quantification.

### Bioinformatic analysis of 16S rRNA gene sequencing

Fastq files of 51 subjects, T2DM (*n* = 17), MetS (*n* = 17), and healthy controls (*n* = 16), were obtained from the National Center for Biotechnology Information (NCBI) Sequence Read Archive, BioProject no. PRJNA819279. Files contained 2×300 bp paired-end reads of the V3–V4 variable regions of the 16S ribosomal RNA (rRNA) gene sequenced from stool samples in Illumina MiSeq System. Raw data analysis was performed using QIIME 2.0 (Quantitative Insights into Microbial Ecology). The DADA2 algorithm was used to remove read sequencing errors and chimeric sequences and to infer amplicon sequence variants [42]. Read quality scores were visualized, and truncation lengths of 220 bp (forward) and 200 bp (reverse) were used. Taxonomic assignments were performed using the Greengenes2 database through the QIIME2 Greengenes2 plugin (q2-greengenes2) [43], following the non-v4-16S pipeline. Taxon bar plots were visualized at view.qiime2.org, and the .csv files containing taxonomic annotations were downloaded.

All of the following analyses were conducted in the R programming language. α-diversity was estimated using the richness, Shannon, Chao1, and Simpson indices calculated from raw count data with the vegan package. Relative abundances were computed by normalizing taxon counts to the total counts per sample. For raw data normalization, 2 different approaches were evaluated. In the first approach, raw counts were normalized using the median-ratio method to estimate size factors with the DESeq2 package. In the second approach, relative abundances were centered log-ratio (CLR) transformed using the composition package, which accounts for the compositional nature of microbiota data [44]. Before CLR transformation, taxa detected in fewer than 10% of the samples at the genus and species levels and 5% at the family level were removed, and zero values in the relative abundance matrix were imputed using the multiplicative replacement method with the zCompositions package.

Beta diversity was assessed using Bray–Curtis dissimilarities calculated from DESeq-normalized counts and visualized with principal coordinates analysis (PCoA) in the vegan package. Differences in community composition among groups were evaluated using permutational multivariate analysis of variance (PERMANOVA) with the adonis2() and pairwise.adonis2() functions, based on the Bray–Curtis dissimilarity matrix and 999 permutations.

### Statistical analysis

Statistical differences in demographic and clinical variables and cytokines among groups were determined using JMP software version 14.0 (SAS Institute, USA). Shapiro–Wilk tests were conducted to assess the normality of each variable, and the Kruskal–Wallis test with Nemenyi’s *post hoc* test was used for nonparametric data, and analysis of variance with Tukey’s *post hoc* test for parametric data.

All of the following statistical analyses were performed using the R programming language. DESeq and CLR-normalized relative abundance data were evaluated to identify significant differences in gut microbiota composition, and taxa with low prevalence (<10% for species and genera and <5% for families) were removed to reduce sparsity and improve statistical power. Significant differences among groups for esterified FAs and bacterial taxa were established using the Kruskal–Wallis test with Dunn’s *post hoc* test. Regression analyses using generalized additive models with the mgcv package evaluated the effects of Tanner stage, age, sex, obesity-related parameters, and metformin on the associations between FAs and microbial taxa and T2DM and MetS (Tables S4 and S13). Nested generalized additive models were compared using analysis of deviance with likelihood ratio tests (χ^2^) and the Akaike information criterion and Bayesian information criterion. Spearman correlations and significance were determined using the cor() and cor.test() functions, respectively.

*P* values were adjusted within each data type (targeted lipidomics or metagenomics) for each statistical analysis using Benjamini and Hochberg’s method [45] to control the false discovery rate (FDR). Statistical analyses and *P*-value adjustments were performed jointly for FAs from both lipidic fractions (PL and CE). For metagenomic data, analyses and *P*-value adjustments were conducted separately at each taxonomic level (phylum, family, genus, and species). Principal component analyses (PCAs) were conducted using the prcomp() function.

## Results

### Clinical characteristics and cytokine levels of the study group

The study enrolled 32 females and 28 males, aged 7 to 17 years (Table 1). All subjects with T2DM were classified as Tanner stages 3 to 5. MetS and healthy groups had 13 subjects in Tanner stages 3 to 5, and in Tanner stages 1 and 2, there were 6 and 8 subjects, respectively. The metabolic risk factors reported by Cook *et al.* [10] for MetS diagnosis, including abdominal obesity (WC), low HDL-c, high TG, and high serum glucose, were significantly different in MetS when compared to those in the healthy group (*P* < 0.05). These variables also differed between T2DM and the controls, and as expected for these children, serum glucose values were significantly higher than those in the MetS and healthy groups. Also, insulin and HOMA-IR were higher in T2DM and MetS than in the control group. In addition, BMI, hip circumference, and WC-to-height ratio were higher in T2DM and MetS. According to age- and sex-specific BMI percentiles, in the T2DM and MetS groups, 70% and 63% of subjects had obesity, respectively, and 20% and 5% were overweight, while healthy controls had normal values. The cytokines interleukin-18 (IL-18), interleukin-8 (IL-8), and monocyte chemoattractant protein-1 (MCP-1) were significantly higher in T2DM than in the healthy group (*P* < 0.05) (Table 2).

**Table 1.** Demographic, anthropometric, and biochemical characteristics of children with type 2 diabetes mellitus (T2DM) and metabolic syndrome (MetS) and a healthy group. Values represent mean ± SD for parametric data and the median and interquartile range for nonparametric data. ANOVA with Tukey’s *post hoc* test was used for parametric data, and Kruskal–Wallis with Nemenyi’s *post hoc* test for nonparametric data. Obesity was defined as BMI ≥95th percentile, and overweight as BMI ≥85th and <95th percentiles [35].

Parameter	T2DM (*n* = 20) ^a^	MetS (*n* = 19)	Healthy (*n* = 21)	*P*
Male	10	10	8	0.851
Female	10	9	13	
Age	14.90 ± 1.52	12.42 ± 2.19	12.48 ± 3.16	0.097
Tanner 1–2	0 ^B^	6 ^A^	8 ^A^	<0.001
Tanner 3–5	20 ^A^	13 ^B^	13 ^B^	
Acanthosis nigricans	19 ^B^	17 ^B^	19 ^A^	0.002
Obesity	14 ^A^	12 ^A^	0 ^B^	<0.001
Overweight	4 ^A^	5 ^A^	0 ^B^	
Normal weight	2 ^B^	2 ^B^	21 ^A^	
Metformin treatment	12 ^A^	12 ^A^	0 ^B^	<0.001
Insulin treatment	2	0	0	0.142
Weight (kg)	68.40 (62.00 – 88.55) ^A^	66.80 (52.75 – 80.20) ^A^	43.90 (30.10 – 54) ^B^	<0.001
Height (cm)	161.15 ± 9.41 ^A^	155.82 ± 12.41 ^B^	149.33 ± 15.28 ^B^	0.015
BMI (kg/m^2^)	27.80 (25.48 – 32.23) ^A^	26.00 (24.00 – 31.55) ^A^	18.00 (17.20 – 21.60) ^B^	<0.001
BMI percentile	96.50 (92.00 – 99.00) ^A^	98.00 (92.50 – 99.00) ^A^	63.00 (42.00 – 70.00) ^B^	<0.001
HipC (cm)	99.00 (90.75 – 112.50) ^A^	94.60 (89.50 – 104.50) ^A^	70.00 (62.50 – 78.30) ^B^	<0.001
WC percentile	85.00 (75.00 – 95.00) ^A^	85.00 (75.00 – 90.00) ^A^	25.00 (25.00 – 25.00) ^B^	<0.001
WC-to-HipC ratio	0.96 (0.90 – 0.97)	0.92 (0.84 – 0.96)	0.96 (0.95 – 0.97)	0.353
WC-to-height ratio	0.59 (0.55 – 0.63) ^A^	0.57 (0.54 – 0.62) ^A^	0.45 (0.44 – 0.46) ^B^	<0.001
SBP percentile	58.00 (34.75 – 88.75)	71.50 (33 – 86)	40.00 (32.50–45)	0.067
DBP percentile	63.65 ± 25.35	58.11 ± 25.91	61.55 ± 11.94	0.737
TC (mg/dl)	149.50 ± 29.24	166.11 ± 39.22	152.29 ± 21.18	0.200
TG (mg/dl)	157.00 (114.25 – 189.75) ^A^	143.00 (118.00 – 208.50) ^A^	87.00 (55.00 – 96.00) ^B^	<0.001
HDL-c (mg/dl)	37.80 ± 7.66 ^B^	39.58 ± 8.21 ^B^	53.43 ± 12.01 ^A^	<0.001
LDL-c (mg/dl)	74.90 ± 20.63 ^B^	94.42 ± 35.51 ^A^	97.38 ± 22.08 ^A^	0.019
Glc (mg/dl)	114.50 (92.00 – 173.75) ^A^	94.00 (88.50 – 99.50) ^B^	87.00 (78.00 – 89.00) ^C^	<0.001
Insulin (mIU/l)	23.30 (11.48 – 30.18) ^A^	19.20 (14.65 – 25.75) ^A^	7.64 (5.78 – 8.82)^B^	<0.001
HOMA-IR	8.37 (3.94 – 12.53) ^A^	4.76 (3.46 – 5.90) ^A^	1.50 (1.06 – 1.89) ^B^	<0.001

ANOVA, analysis of variance; BMI, body mass index; WC, waist circumference; HipC, hip circumference; SBP, systolic blood pressure; DBP, diastolic blood pressure; TC, total cholesterol; TG, triglycerides; HDL-c, high-density lipoprotein cholesterol; LDL-c, low-density lipoprotein cholesterol; Glc, fasting glucose; HOMA-IR, homeostatic model assessment of insulin resistance

^a^ Groups labeled with different letters within each variable are significantly different (*P* < 0.05).

**Table 2.** Plasma cytokine levels in children with type 2 diabetes mellitus (T2DM) and metabolic syndrome (MetS) and a healthy group. Values represent mean ± SD for parametric data and the median and interquartile range for nonparametric data. ANOVA with Tukey’s *post hoc* test was used for parametric data, and Kruskal–Wallis with Nemenyi’s *post hoc* test for nonparametric data.

Cytokine (pg/ml)	T2DM (*n* = 18) ^a^	MetS (*n* = 17)	Healthy (*n* = 16)	*P*
IL-1β	0.89 (0.54 – 2.71)	0.57 (0.4 – 1.3)	0.78 (0.13 – 1.84)	0.477
INF-α2	1.29 (0.21 – 5.31)	3.84 (0.48 – 6.74)	0.38 (0.11 – 2.81)	0.086
INF-γ	4.10 (2.06 – 5.3)	3.54 (2.22 – 4.36)	2.23 (0.27 – 4.28)	0.152
TNF-α	1.43 (0.83 – 1.91)	1.69 (1.07 – 2.06)	1.43 (0.47 – 2.52)	0.786
MCP-1	254.97 ± 70.54 ^A^	221.08 ± 65.76 ^B^	197.21 ± 38.64 ^B^	0.023
IL-6	4.05 ± 2.07	3.56 ± 1.62	2.88 ± 1.77	0.177
IL-8	8.16 (6.27 – 12.04) ^A^	5.99 (3.47 – 8.6) ^B^	4.64 (2.78 – 6.1) ^B^	0.023
IL-10	2.23 ± 1.01	1.64 ± 0.81	1.61 ± 1.13	0.106
IL-12p70	0.57 (0.24 – 1.12)	0.48 (0.21 – 1.11)	0.42 (0.14 – 0.73)	0.629
IL-17A	6.72 (4.94 – 15.14)	10.9 (5.14 – 18.71)	6.70 (1.5 – 11.41)	0.28
IL-18	224.26 (183.94 – 272.78) ^A^	203.01 (125.23 – 246.08) ^B^	139.59 (108.77 – 175.91) ^B^	0.002
IL-23	5.88 (2.65 – 7.5)	4.42 (3 – 7.79)	4.39 (2.45 – 6)	0.625
IL-33	10.53 (5.29 – 15.13)	7.25 (4.4 – 19)	8.25 (2.45 – 11.65)	0.505

ANOVA, analysis of variance; IL, interleukin; INF, interferon; TNF-α, tumor necrosis factor-α; MCP-1, monocyte chemoattractant protein-1

^a^ Groups labeled with different letters within each variable are significantly different (*P* < 0.05).

### Clustering of T2DM and MetS subjects by patterns of esterified FA concentrations

**Fig. 2.**
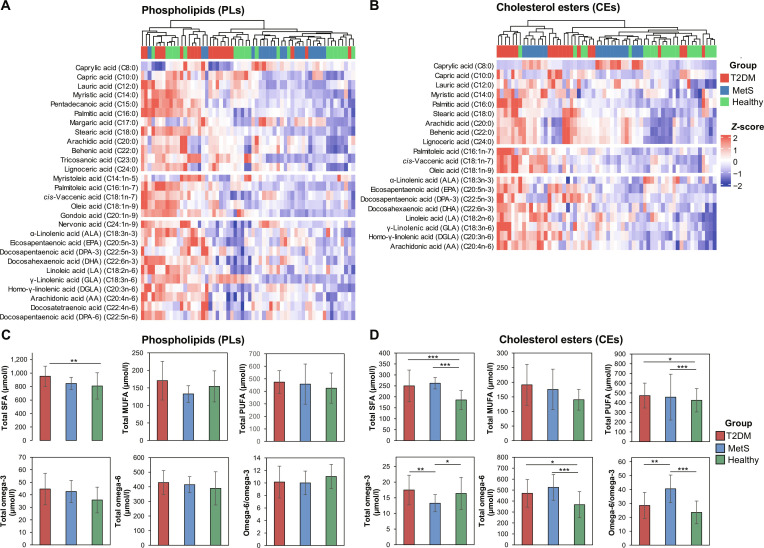
Fatty acids (FAs) esterified to plasma phospholipids (PLs) and cholesterol esters (CEs) in children and adolescents with type 2 diabetes mellitus (T2DM) and metabolic syndrome (MetS) and a healthy group. (A and B) Heatmaps of the *z*-score values of FA concentrations. Color represents intensity ranges from higher (red) to lower (blue) values. (C and D) Total saturated FAs (SFAs), monounsaturated FAs (MUFAs), polyunsaturated FAs (PUFAs), omega-3 and omega-6 FAs, and omega-3/omega-6 ratio. Asterisks indicate significant differences (Kruskal–Wallis with Dunn’s *post hoc* test): false discovery rate (FDR) *P* < 0.05 (*); FDR *P* < 0.01 (**); FDR *P* < 0.001 (***).

Concentrations of FAs esterified to plasma PLs and CEs in children and adolescents with T2DM and MetS and in healthy controls showed significant differences among groups. Dendrograms of heatmaps in Fig. 2A and B showed distinctive subject clusters, even within the groups, based on the patterns of FA concentrations (*z*-scores). The PCA biplot shown in Fig. S1 included significantly different PL-FAs and explained 48.1% of the total variance by PC1, separating most subjects with T2DM from those with MetS. PC2 accounted for 20.2% of the variance and distinguished MetS from healthy controls. Similarly, in the PCA biplot for CE-FAs, PC1 explained 39.9% of the variance. It separated children with T2DM from controls, whereas PC2 accounted for 16.5% of the variance, discriminating MetS from the other 2 groups.

### Differential FAs esterified to plasma PLs and CEs in pediatric T2DM and MetS

Palmitic acid (C16:0, 298 to 551 μmol/l), stearic acid (C18:0, 200 to 355 μmol/l), and LA (C18:2n-6, 174 to 321 μmol/l) were present at the highest concentrations in the PL fraction (Table S1). The sum of PL-saturated FAs (SFAs) was significantly higher in T2DM than in the healthy group (FDR *P* < 0.05), while no differences were observed for total monounsaturated FAs (MUFAs), polyunsaturated FAs (PUFAs), omega-3, and omega-6 FAs in PLs (Fig. 2C).

**Fig. 3.**
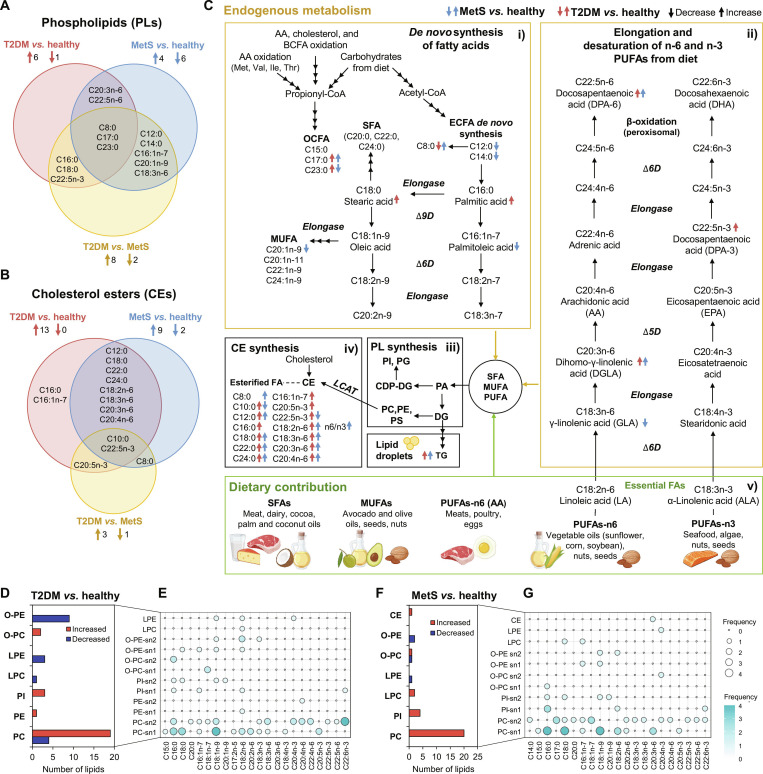
Differential fatty acids (FAs) esterified to plasma phospholipids (PLs) and cholesterol esters (CEs) in children and adolescents with type 2 diabetes mellitus (T2DM) and metabolic syndrome (MetS), compared to those in the healthy group. (A and B) Venn diagrams of significantly different FAs among groups (Kruskal–Wallis with Dunn’s *post hoc* test, false discovery rate [FDR] *P* < 0.05). (C) Schematic representation of endogenous metabolism and exogenous sources of FAs: (i) *De novo* synthesis of even-chain FAs (ECFAs) and odd-chain FAs (OCFAs). (ii) Elongation and desaturation of omega-3 and omega-6 FAs. Red and blue arrows in panels (i) and (ii) show significant FAs in the PL fraction. (iii) PL synthesis, where the FA pool is used to form glycerophosphate (PA). (iv) CE synthesis, in which the FA in the sn-2 position of a PL is transferred to free cholesterol. (v) Main dietary sources of FAs [72]. (D and F) Number of significant PLs and CEs from the untargeted lipidomics previously reported with a subgroup of subjects (*n* = 30) [24]. (E and G) Esterified FA frequency in significant PLs and CEs. SFA, saturated FA; MUFA, monounsaturated FA; PUFA, polyunsaturated FA; PI, phosphoinositol; PG, phosphoglycerol; PC, phosphocholine; PE, phosphoethanolamine; PS, phosphoserine. LCAT, lecithin:cholesterol acyl transferase; Δ5D, delta-5-desaturase; Δ6D, delta-6-desaturase; Δ9D, delta-9-desaturase.

Distinctive differential PL-SFAs among all groups were caprylic acid (C8:0), heptadecanoic acid (C17:0), and tricosanoic acid (C23:0), as shown in the Venn diagram and schematic representation of FA biosynthesis (Fig. 3A and C and Table S1). MetS showed the highest plasma concentrations of PL-C8:0 and PL-C17:0. These FAs were lower and higher, respectively, in T2DM compared to those in controls. The T2DM group had the highest levels of PL-C23:0, followed by healthy and MetS subjects. Additionally, in the PL fraction, palmitic acid (C16:0), stearic acid (C18:0), and docosapentaenoic acid (DPA-3; C22:5n-3) were augmented in T2DM versus MetS and healthy groups. In the MetS group, lauric acid (C12:0), myristic acid (C14:0), palmitoleic acid (C16:1n-7), gondoic acid (C20:1n-9), and γ-linolenic acid (GLA; C18:3n-6) were diminished when compared to those in the other groups (FDR *P* < 0.05). The PL-omega-6 FAs, DGLA (C20:3n-6) and docosapentaenoic acid (DPA-6; C22:5n-6), increased in both T2DM and MetS versus the healthy group.

The highest concentrations of FAs esterified to cholesterol were LA (C18:2n-6, 211 to 570 μmol/l), oleic acid (C18:1n-9, 85 to 213 μmol/l), and palmitic acid (C16:0, 68 to 202 μmol/l) (Table S1). The sums of FAs indicated that total CE-SFAs, PUFAs, and omega-6 FAs were significantly higher in T2DM and MetS compared to those in the healthy group. Total omega-3 FAs were lower in MetS, increasing the CE-omega-6/omega-3 ratio (FDR *P* < 0.05) (Fig. 2D). In the CE fraction, capric acid (C10:0) and DPA-3 (C22:5n-3) were significantly different among all groups (Fig. 3B and C and Table S1). Also, T2DM and MetS showed higher levels of 4 CE-SFAs, lauric acid (C12:0), stearic acid (C18:0), behenic acid (C22:0), and lignoceric acid (C24:0), and 4 CE-omega-6 FAs, LA (C18:2n-6), GLA (C18:3n-6), DGLA (C20:3n-6), and arachidonic acid (AA; C20:4n-6), than the healthy controls. Additionally, the T2DM group presented increased concentrations of C16:0, C16:1n-7, and EPA-3 (C20:5n-3), and MetS of C8:0 (FDR *P* < 0.05).

### Effect of pubertal development and obesity on the association of esterified FAs with pediatric T2DM and MetS

The effect of pubertal development on the association between esterified FAs and T2DM or MetS was evaluated using regression models adjusted for Tanner stage or/and age and sex, with the healthy group as reference (Tables S2 to S4). Likelihood ratio tests showed no significant differences between the models (*P* > 0.05). The model adjusted for Tanner and sex was selected since it showed the best balance between model fit and complexity (lowest Akaike and Bayesian information criterion values) (Table S4). In addition, regression coefficients (*β*) and the overall pattern of significant associations were consistent across models (Tables S2 and S3).

**Fig. 4.**
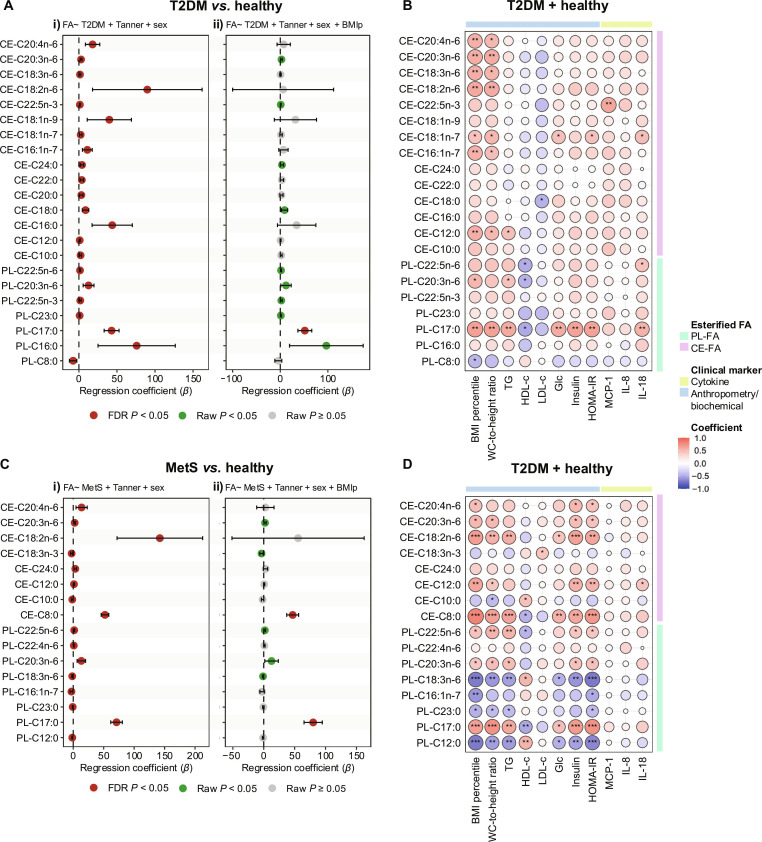
Association of fatty acids (FAs) esterified to plasma phospholipids (PLs) and cholesterol esters (CEs) with clinical and inflammatory biomarkers in children and adolescents with type 2 diabetes mellitus (T2DM) and metabolic syndrome (MetS). (A and C) Forest plots showing the regression coefficient (*β*) with 95% confidence interval (CI) of FAs in (A) T2DM and (C) MetS, in reference to the healthy group: (i) Models adjusted for Tanner stage and sex. (ii) Models adjusted for Tanner stage, sex, and body mass index percentile (BMIp). (B and D) Spearman correlation coefficients of FAs with clinical and inflammatory markers. Correlations in T2DM and MetS also included healthy subjects. Dot size and color intensity represent the coefficient (red = positive; blue = negative). Asterisks indicate significant correlations: false discovery rate (FDR) *P* < 0.05 (*); FDR *P* < 0.01 (**); FDR *P* < 0.001 (***). BMI, body mass index; WC, waist circumference; TG, triglycerides; HDL-c, high-density lipoprotein cholesterol; LDL-c, low-density lipoprotein cholesterol; Glc, fasting glucose; HOMA-IR, homeostatic model assessment of insulin resistance; MCP-1, monocyte chemoattractant protein-1; IL, interleukin.

The forest plots in Fig. 4A and C (i) show the FAs that remained significantly associated with T2DM or MetS after adjustment for Tanner stage and sex. Adjustment for these covariates affected the associations of PL-C18:0 and CE-C20:5n-3 with T2DM and PL-C8:0, PL-C14, PL-C20:1n-9, CE-C18:0, CE-22:0, CE-C18:3n-6, and CE-C22:5n-3 with MetS, which were no longer significant. Conversely, adjustment revealed significant associations for CE-C20:0, CE-C18:1n-7, and CE-C18:1n-9 with T2DM and for PL-C22:4n-6 and CE-C18:3n-3 with MetS.

The model evaluating the effect of obesity (BMI percentile) in Fig. 4A and C (ii) shows a decrease in significance for most FAs, except for the associations of PL-C17:0 with T2DM and PL-C17:0 and CE-C8:0 with MetS (FDR *P* < 0.05). Additionally, the regression coefficients (*β*) of PL-C17 increased for both T2DM (from 42.94 to 51.55) and MetS (from 71.06 to 79.60), while for CE-C8:0, the *β* value decreased from 52.10 to 46.78.

### Correlation of esterified FAs with anthropometric, biochemical, and inflammatory biomarkers

Spearman correlations assessed the association between esterified FAs and anthropometric, biochemical, and inflammatory biomarkers for each study group (Fig. 4B and D). The levels of PL-C17 in the T2DM and MetS groups, including healthy controls, directly correlated with obesity-related parameters (BMI percentile and WC-to-height ratio), TG, glucose, and HOMA-IR, and in T2DM, with the cytokine IL-18 (FDR *P* < 0.05). In contrast, this FA negatively correlated with HDL-c in both groups. Additionally, in T2DM, most omega-6 FAs from PL and CE fractions, CE-MUFAs (C16:1n-7 and C18:1n-7), and CE-C12:0 positively correlated with BMI percentile and WC-to-height ratio (Fig. 4B). In MetS, including healthy subjects, CE-omega-6 FAs, PL-C20:3n-6, CE-C8:0, and CE-C12:0 directly correlated with obesity-related parameters, TG, and altered glucose homeostasis (FDR *P* < 0.05) (Fig. 4D). In contrast, PL-C12:0, PL-C23:0, PL-C16:1n-7, and PL-C18:3n-6 presented negative correlations with the same clinical markers.

### Differential gut microbiota composition in pediatric T2DM and MetS

**Fig. 5.**
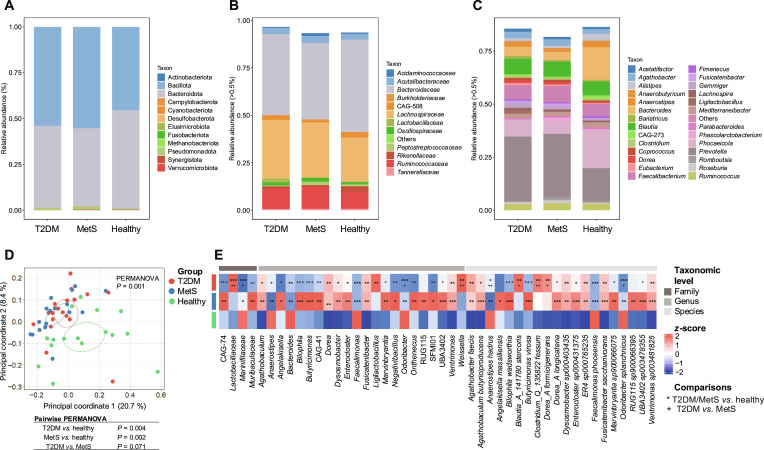
Gut microbiota composition in children and adolescents with type 2 diabetes mellitus (T2DM) and metabolic syndrome (MetS) and a healthy group. (A to C) Relative abundance of bacteria at the (A) phylum, (B) family, and (C) genus levels. Values represent the average across all samples. (D) Principal coordinates analysis (PCoA) at the genus level and permutational multivariate analyses of variance (PERMANOVAs). (E) Heatmap of the *z*-score values of DESeq2-normalized counts, showing significantly different bacterial taxa among groups (Kruskal–Wallis with Dunn’s *post hoc* test, false discovery rate [FDR] *P* < 0.05). Color represents intensity ranges from higher (red) to lower (blue) values. Symbols indicate significant differences between T2DM or MetS and the healthy group (*) and between the T2DM and MetS groups (+): FDR *P* < 0.05 (* or +); FDR *P* < 0.01 (** or ++); FDR *P* < 0.001 (*** or +++).

In this study, we analyzed the 16S rRNA gene sequencing data of 51 subjects, previously reported for the cohort [32], using an updated version of the Greengenes database (Greengenes2). This improved tool enabled a higher number of taxonomic annotations and more taxa with significant changes in pediatric groups with metabolic diseases (Fig. 5). The dominant phyla on average across all samples were Bacillota (51.1% ± 24.1%) and Bacteroidota (46.9% ± 24.6%), with a ratio (Bacillota/Bacteroidota) resulting in 9.1 ± 31.4 for T2DM, 5.3 ± 13.5 for MetS, and 1.5 ± 1.5 for the healthy group (Fig. 5A and Fig. S4). The most prevalent taxa at the family level were *Bacteroidaceae* (43.8% ± 24.5%) and *Lachnospiraceae* (27.91% ± 12.7%) and at the genus level were *Prevotella* (26.7% ± 26.8%), *Phocaeicola* (10.3% ± 12.6%), and *Bacteroides* (7.7% ± 11.2%) (Fig. 5B and C and Figs. S5 and S6). The most abundant species were *Prevotella copri* (25.0% ± 26.5%), *Phocaeicola_A_858004 vulgatus* (8.4% ± 11.8%), *Faecalibacterium prausnitzii_C_71358* (6.6% ± 8.8%), and *Bacteroides_H stercoris* (6.6% ± 7.3%) (Fig. S7).

Nonsignificant differences were observed in α-diversity indices among groups; however, MetS showed higher average values for Shannon, Chao1, and richness indices than T2DM and healthy controls (Fig. S3). β-diversity differed between the T2DM/MetS and healthy groups at the genus level (PERMANOVA, *P* < 0.05). PCoA plot clustered T2DM and MetS apart from healthy controls (Fig. 5D). A total of 51 significantly different taxa among groups were identified when using DESeq-normalized counts (Fig. 5E and Table S5), and 15 taxa with CLR-transformed abundances (Table S6). The consistently significant taxa in both datasets were 3 families (CAG-74, *Lactobacillaceae*, and *Marinifilaceae*), 5 genera (*Faecalimonas*, *Odoribacter*, *Ventrimonas*, *Weissella*, and an unknown genus), and 5 species (*Faecalimonas phoceensis*, *Fusicatenibacter saccharivorans*, *Odoribacter splanchnicus*, *Ventrimonas* sp003481825, and an unknown species) (FDR *P* < 0.05).

According to DESeq-normalized counts, at the family level, *Lactobacillaceae* significantly increased in T2DM, and CAG-74 and *Muribaculaceae* in MetS, and *Marinifilaceae* decreased in both groups, compared with that in the healthy controls (Fig. 5E). In addition, 8 bacterial genera were higher and 4 were lower in T2DM than in the healthy controls. In MetS, a greater number of microbial taxa showed significant differences; 16 genera were higher and 4 were lower than in the healthy group. The common genera that significantly increased in T2DM and MetS were *Agathobaculum*, *Dorea*, *Dysosmobacter*, *Enterocloster*, *Fusicatenibacter*, and *Ventrimonas*. In contrast, *Anaerostipes*, *Bacteroides*, *Faecalimonas*, and *Odoribacter* decreased in both groups.

### Effect of pubertal development and obesity on the association of gut microbial taxa with pediatric T2DM and MetS

**Fig. 6.**
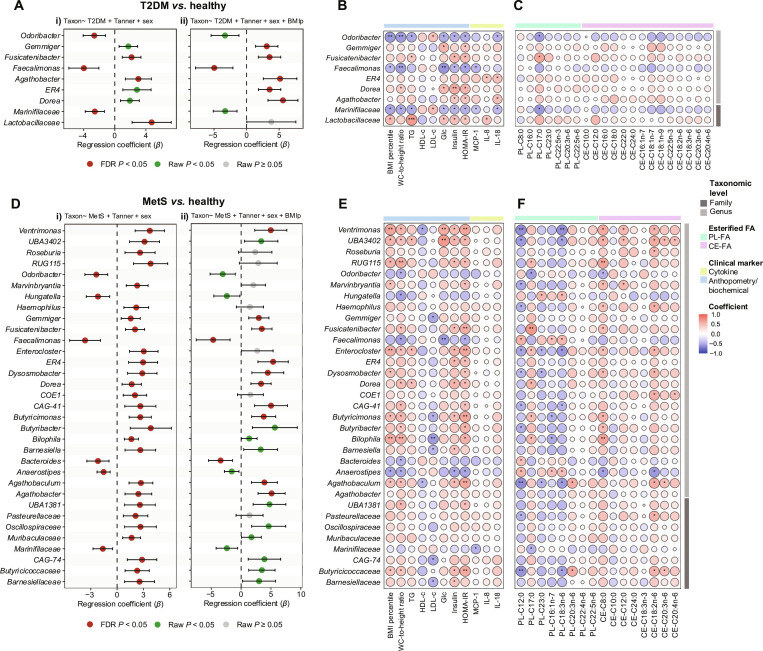
Association of gut microbial taxa with clinical and inflammatory markers and fatty acids (FAs) esterified to plasma phospholipids (PLs) and cholesterol esters (CEs) in children and adolescents with type 2 diabetes mellitus (T2DM) and metabolic syndrome (MetS). (A and D) Forest plots showing the regression coefficient (*β*) with 95% confidence interval (CI) of microbial taxa in (A) T2DM and (B) MetS, in reference to the healthy group: (i) Models adjusted for Tanner stage and sex. (ii) Models adjusted for Tanner stage, sex, and body mass index percentile (BMIp). (B and E) Spearman correlation coefficients of bacterial taxa with clinical markers. (C and F) Spearman correlation coefficients of bacterial taxa with FAs. Correlations in (B and C) T2DM and (E and F) MetS also included healthy subjects. Dot size and color intensity represent the coefficient (red = positive; blue = negative). Asterisks indicate significant correlations: false discovery rate (FDR) *P* < 0.05 (*); FDR *P* < 0.01 (**); FDR *P* < 0.001 (***). BMI, body mass index; WC, waist circumference; TG, triglycerides; HDL-c, high-density lipoprotein cholesterol; LDL-c, low-density lipoprotein cholesterol; Glc, fasting glucose; HOMA-IR, homeostatic model assessment of insulin resistance; MCP-1, monocyte chemoattractant protein-1; IL, interleukin.

Regression models adjusted for Tanner stage and sex alone, or including age, evaluated the effect of pubertal development on the association between microbial taxa and T2DM or MetS (Tables S7 to S13). Like the findings for plasma FAs, no significant differences were detected between models (likelihood ratio test, *P* > 0.05), and the microbial associations remained consistent. The forest plots in Fig. 6A and D (i) show the microbial taxa that remained significantly associated with T2DM or MetS after adjustment for Tanner stage and sex. The associations of the bacterial families *Lactobacillaceae* and *Marinifilaceae* and the genera *Agathobacter*, *Faecalimonas*, *Fusicatenibacter*, and *Odoribacter* with T2DM were independent of pubertal development and sex (FDR *P* < 0.05) (Fig. 6A (i)). These associations also remained significant after additional adjustment for obesity, except for the family *Lactobacillaceae* and the genus *Odoribacter* (Fig. 6A (ii)).

In MetS, 7 microbial families and 25 genera were associated with the group condition after Tanner stage and sex adjustment (FDR *P* < 0.05) (Fig. 6D (i)). Of these, only 1 family and 5 genera showed a negative regression coefficient. The models additionally adjusted for BMI percentile showed a decrease in the significance for all of the families and 13 of the genera previously associated with MetS, while the patterns in the regression coefficients did not change (Fig. 6D (ii)).

### Correlation of gut microbiota composition with clinical and inflammatory biomarkers and esterified FAs

Spearman correlations in the T2DM, including the healthy subjects, showed positive correlations between the bacterial family *Lactobacillaceae* and BMI percentile, TG, glucose, insulin, HOMA-IR, and cytokine IL-8 (Fig. 6B). In contrast, the family *Marinifilaceae* and the genera *Faecalimonas* and *Odoribacter* negatively correlated with most of these clinical variables, as well as with WC-to-height ratio and the cytokines MCP-1 and IL-8 (FDR *P* < 0.05). Additionally, higher abundances of the genera *Agathobacter*, *Dorea*, *Fusicatenibacter*, and *Gemmiger* directly correlated with altered glucose homeostasis parameters (glucose, insulin, and HOMA-IR) in the T2DM plus healthy subject dataset. In these subjects, only PL-C17:0 showed positive correlations with *Fusicatenibacter* and negative correlations with *Marinifilaceae* and the genus *Odoribacter* (FDR *P* < 0.05) (Fig. 6C).

In MetS, including healthy controls, the family *Butyricicoccaceae* and genera *Agathobaculum*, *Bilophila*, *Butyricibacter*, *Butyricimonas*, *Dorea*, *Dysosmobacter*, *Enterocloster*, *Fusicatenibacter*, *Marvinbryantia*, RUG115, UBA3402, and *Ventrimonas* positively correlated with obesity-related parameters (BMI percentile or WC-to-height ratio) and increased glucose, insulin, and HOMA-IR (FDR *P* < 0.05) (Fig. 6E). Several of these microbial genera directly correlated with PL-C17:0, CE-C8:0, or C18:2n-6 (Fig. 6F).

## Discussion

### Clinical metabolic risk factors and inflammatory biomarkers characterizing the study groups

Youth-onset T2DM presents an aggressive clinical course, and it is often preceded by obesity and MetS [1,3–5,46]. In our study, all children and adolescents with T2DM and MetS were overweight or had obesity. They presented several cardiometabolic risk factors defined by Cook *et al.* [10] for MetS diagnosis, including dyslipidemia, insulin resistance, and abdominal obesity (Table 1). In addition, the pro-inflammatory cytokines MCP-1, IL-8, and IL-18 were elevated in T2DM and, to a lesser extent, in MetS compared with those in the healthy group (Table 2). These observations are consistent with previous findings from the same cohort, in which MCP-1 and IL-18 showed positive correlations with adhesion molecules, suggesting endothelial dysfunction, and negative correlations with irisin, an adipomyokine linked to improving insulin sensitivity and anti-inflammatory effects [9].

### Association of FAs esterified to PL and cholesterol metabolism

Changes in esterified plasma FAs have been characterized in obesity-derived comorbidities and associated with altered glucose homeostasis and low-grade inflammation in children and adolescents [26,47] and adults [16,25]. In a previous nonquantitative lipidomic study, with a subgroup of subjects (*n* = 30) from the present cohort, we observed several PL classes associated with T2DM and MetS [24]. Specifically, phosphatidylcholines and phosphoinositols were higher, and LysoPLs and plasmalogens were lower in these metabolic conditions and showed unique esterified FAs (Fig. 3D to G).

PLs are the first reservoir of FAs from endogenous metabolism, including *de novo* lipogenesis, desaturation, elongation, retroconversion, and oxidation of FAs, and exogenous FAs from dietary intake [25,27], as shown in Fig. 3C. PLs usually feature an SFA or a MUFA in the sn-1 position and a PUFA in the sn-2 [27]. Accordingly, quantification of the FAs in the plasma of pediatric patients from the present study indicated that SFAs were the most abundant in the PL fraction. The highest concentrations were observed for C16:0 and C18:0, followed by omega-6 PUFAs with C18:2n-6 and C20:4n-6 as the most predominant (Table S1). These results were consistent with previous studies in adults and children [25–27,47,48].

The sn-2 FA in PLs is transferred to free cholesterol on the surface of HDL-c to form CE by the enzyme LCAT [21,22]. Higher LCAT activity has been linked to protection against atherosclerosis, since when free cholesterol is transformed to CE, it migrates to the core of HDL-c particles, contributing to HDL-c maturation and reverse cholesterol transport [21–23]. Therefore, the type of FA bound to the CE lipid could affect HDL-c functionality. Thus, the characterization of FAs in CEs reflects the available FA pool in PLs and LCAT substrate preference. In the CE fraction, PUFAs were the most abundant FAs, with C18:2n-6 exhibiting the highest concentrations (Table S1), in agreement with previous studies [21,26,27]. Moreover, both prior studies [26,27] and our findings identified C18:1n-9 and C16:0 as the next most prevalent FAs in CEs. Figure S2 shows a correlation matrix between PL-FAs and CE-FAs, which was used to explore LCAT preference for specific FAs. The FAs C8:0, C16:0, C16:1n-7, C18:1n-9, C18:3n-3, C20:5n-3, C22:6n-3, and C20:3n-6 increased in CEs as they increased in PLs, while levels of C18:3n-6 in CEs were higher at lower levels in PLs, suggesting that C18:3n-6 is mainly accumulated in the CE fraction.

On the other hand, lower LCAT activity has been associated with higher levels of pro-inflammatory FAs in children and adolescents with abdominal obesity [22]. Additionally, in adults with T2DM, LCAT activity decreased and was directly correlated with BMI [21]. In our study, HDL-c was lower in T2DM and MetS, and although there were nonsignificant differences in TC, values were higher on average in MetS, suggesting a disrupted cholesterol transport.

### Elevated esterified omega-6 FAs characterized pediatric T2DM and MetS

In the clinical context, obesity and MetS can precede T2DM [1,3–5,46]. Therefore, associations of FAs with T2DM and MetS pathophysiology are likely influenced by the distinct metabolic alterations as defined by the MetS diagnosis criteria [10]. However, as the disease progresses to T2DM, various compensatory or impaired lipid-metabolic mechanisms may be activated. On the other hand, plasma FA levels have been correlated with dietary FA intake [27].

In this study, we identified several esterified FAs that were commonly altered in both the T2DM and MetS groups compared to those in healthy controls. These included mainly SFAs and omega-6 FAs (Figs. 3 and 4). Specifically, in the CE fraction, LA (C18:2n-6) levels were significantly higher in the T2DM and MetS groups, whereas α-linoleic acid (ALA; C18:3n-3) concentrations were lower in MetS. Since LA and ALA are essential FAs obtained exclusively from diet, these findings may reflect a dietary pattern characterized by higher LA and lower ALA intakes in children with T2DM and MetS. Additionally, DGLA (C20:3n-6) in both the PL and CE fractions, as well as PL-DPA-6 (C22:5n-6) and CE-AA (C20:4n-6), were also elevated in subjects with T2DM and MetS, along with an increased CE-omega-6/omega-3 ratio in MetS. In a previous study, dietary LA and AA intakes were positively correlated with their respective concentrations in plasma PL and CE fractions and whole plasma, while DGLA concentrations showed no such association [27].

Increased omega-6 FAs were directly correlated with obesity-related parameters (BMI percentile and WC-to-height ratio) in T2DM and MetS and insulin resistance in MetS (Fig. 4). Previous studies have also linked higher levels of omega-6 FAs in the PL fraction, including DGLA (C20:3n-6) and DPA-6 (C22:5n-6), with an increased incidence of T2DM in adults [16]. Likewise, a higher omega-6/omega-3 ratio has been associated with dyslipidemia, showing positive correlations with TG, TC, LDL-c, TC/HDL-c ratio, and apolipoprotein B (ApoB) [49]. In a pediatric cohort with obesity and MetS, the plasma concentrations of PL-LA (C18:2n-6) were lower than those in normal-weight subjects, while PL-DGLA (C20:3n-6) levels were higher and directly associated with increased abdominal obesity (WC), HOMA-IR, and TG. However, in this previous study, diet consumption did not explain the associations between differential FAs and obesity and MetS [26].

Additionally, in our study, model adjustment for obesity-related variables attenuated the associations of several omega-6 FAs (PL- and CE-DGLA, PL-DPA-6, CE-LA, and CE-AA) with pediatric T2DM, as evidenced by reduced statistical significance and smaller regression coefficients (Table S2). In MetS, the associations with omega-6 FAs were also attenuated after adjustment for BMI percentile, HOMA-IR, and metformin treatment, suggesting that these factors partially explain the observed associations (Table S3).

Omega-3 FAs are mainly precursors of anti-inflammatory oxylipins, while omega-6 FAs can serve as precursors of pro-inflammatory or anti-inflammatory oxylipins depending on the specific FA [50]. Specifically, DGLA (C20:3n-6) is a precursor of 1-series prostaglandins and thromboxanes, which exert anti-inflammatory effects [26,50]. Bermúdez-Cardona *et al.* [26] explained that elevated DGLA could indicate compensatory mechanisms in children driven by Δ-6-desaturase activity. In T2DM, this could also be suggested for the increased levels of PL-DPA-3 (C22:5n-3), a precursor of pro-resolving (anti-inflammatory) lipid mediators, D-series protectins, and resolvins, and CE-EPA (C20:5n-3), a precursor of 3-series prostaglandins and thromboxanes and E-series resolvins [50].

### Elevated esterified SFAs characterized pediatric T2DM and MetS

Elevated plasma levels of SFAs also characterized pediatric T2DM and MetS. In the T2DM group, even-chain SFAs (ECFAs) containing 10 to 24 carbons in the CE fraction, and in the PL fraction PL-C16:0, PL-C17:0, and PL-C23:0, were increased, whereas PL-C8:0 was decreased. Among these SFAs, PL-C17:0, CE-C12:0, and CE-C24:0 were also elevated in the MetS group, together with CE-C8:0. In adults, higher levels of PL-ECFAs (C14:0, C16:0, and C18:0) were associated with increased risk of coronary heart disease, while odd-chain FAs (OCFAs; C15:0 and C17:0) in PLs showed the contrary [51]. Similarly, in adults, Zheng *et al.* [47] reported associations of increased PL-ECFA (C14:0, C16:0, and C18:0) with higher LDL-c, TC/HDL-c ratio, TG, ApoB, and hepatic biomarkers and lower HDL-c, but inverse associations with PL-OCFA (C15:0 and C17:0). OCFAs have been linked to lower risk of cardiovascular disease, anti-inflammatory activity, and improved glucose and cholesterol levels [52,53]. Very long-chain FAs such as C23:0 and C25:0 represent 10% of adult brain glycosphingolipids, and their synthesis might occur from the shorter OCFAs [52], whereas interventions with medium-chain FAs, such as C8:0, have reported potentiation of insulin secretion [54,55].

In the present study, PL-C17:0 was the only SFA that remained significantly associated with both T2DM and MetS, and C8:0 with MetS across all adjusted regression models (Tables S2 and S3), suggesting that its association is independent of obesity, insulin resistance, and metformin treatment. Elevated plasmatic levels of PL-C17:0 in both groups and CE-C8:0 in MetS were directly correlated with BMI percentile, WC-to-height ratio, TG, glucose, insulin, and HOMA-IR (Fig. 4B and D). Our results suggest that in children and adolescents, C17:0 and C8:0 associations with obesity-linked comorbidities such as T2DM or MetS might differ from those in adults, perhaps by different regulatory mechanisms or dietary intake. Interestingly, the associations of CE-C16:1n-7, CE-C18:1n-7, and CE-C18:1n-9 with pediatric T2DM became evident only after adjustment for Tanner stage and sex, suggesting that differences in pubertal development and sex partially masked these associations in the unadjusted analyses.

### Endogenous and exogenous sources potentially affecting the plasma levels of esterified FAs in pediatrics

The present observational study aimed to quantify FAs esterified to plasma PL and CE fractions. These FA patterns reflect a balance that can be influenced by dietary intake, endogenous metabolism, and, potentially, FAs produced by the gut microbial community. Because of the study design limitations, the individual contributions of endogenous and exogenous sources to plasma FA levels cannot be determined. However, as described in this section, based on scientific knowledge, the differential FA patterns observed in the children and adolescents with T2DM and MetS likely reflect both endogenous metabolism and dietary intake, which together contribute to characteristic plasma lipid profiles.

Results suggested that *de novo* synthesis or dietary intake of FAs was probably higher in T2DM and MetS, evidenced by several SFAs increased in these conditions (Figs. 3 and 4). When the cells sense high concentrations of carbohydrates, these are converted into FA via acetate to produce C16:0-CoA [56]. On the other hand, OCFAs, like PL-C17:0, are *de novo* synthesized from propionyl-CoA, which can be produced from the oxidation of amino acids (methionine, valine, isoleucine, and threonine), cholesterol, and branched-chain FAs, as well as from carbohydrates in the diet [52].

The increase in SFAs could also be due to the preference for omega-6 FAs of the desaturation and elongation systems. The conversion of FAs involves Δ-6- and Δ-5-desaturase activities within the omega-6 pathway, Δ-9- and Δ-6-desaturases in SFA (*de novo* synthesis pathway), and elongase enzymes (Fig. 3C). Park *et al.* [56] observed that cells in the presence of a high concentration of LA (C18:2n-6) decreased the conversion of C16:0 to C16:1, while the desaturation-elongation product C20:3n-6 increased. Accordingly, we observed that in T2DM and MetS, the essential omega-6 FA, C18:2n-6, accumulated in CE fractions, as well as elongated omega-6 FAs with a higher number of unsaturations (e.g., PL- and CE-C20:3n-6, PL-C22:5n-6, and CE-C24:5n-6). The MetS group also featured lower concentrations of PL-MUFAs (C16:1n-7 and C20:1n-9).

Although dietary intake was not assessed in the present study, our findings may be consistent with a higher intake of omega-6 FAs and SFAs, particularly among children with T2DM and MetS. In Mexican children and adolescents, total dietary fat contributes approximately 25% to 27% of total energy intake, with SFAs accounting for 11%, MUFAs for 9%, and PUFAs for approximately 6%, of which about 3% corresponds to omega-6 FAs and only 0.02% to omega-3 FAs [57]. This dietary pattern is characterized by a markedly higher intake of omega-6 than omega-3 FAs, which may contribute to the elevated omega-6 FA levels observed in our cohort. Supporting the influence of diet on FA profiles, a study in children aged 4 to 7 years found that greater consumption of fish, shellfish, nuts, and leafy vegetables was positively associated with omega-3 PUFAs and negatively associated with SFAs. These dietary patterns were also associated with a lower risk of being overweight [58].

### Association of gut microbiota composition with esterified FAs in pediatric T2DM and MetS

Interactions between the gut microbiota and host lipid metabolism may involve microbial lipid transformation, production of short-chain FAs and complex lipids, and modulation of inflammatory pathways linked to metabolic dysfunction [28–30]. Significantly associated microbial taxa with T2DM and MetS after Tanner, sex, and BMI percentile adjustment were *Agathobacter*, *Dorea*, and *Fusicatenibacter* (*F. saccharivorans*), with higher abundances than those in healthy controls, and the genus *Faecalimonas* at lower abundances (Fig. 6A and D). All of these genera belong to the family *Lachnospiraceae*, one of the most predominant families in the Bacillota phylum [59]. A lower abundance of the family *Lachnospiraceae* has been reported in children with obesity and was inversely correlated with energy intake [60]. However, in children and adults with obesity, increased levels of *Lachnospiraceae_bacterium_TF09-5* [14] and the family *Lachnospiraceae* [61], respectively, have also been reported. In contrast, adults with insulin resistance exhibited lower levels of *Lachnospiraceae bacterium*, which were associated with lower fiber intake and higher consumption of refined grains and sugars [62]. *Lachnospiraceae* includes butyrate-producing species, mainly associated with beneficial effects, as well as acetate- and propionate-producing species, whose overproduction has been linked to obesity-related diseases [59,61].

Among the differential microbial taxa in both the T2DM and MetS groups, *Fusicatenibacter* was directly correlated with higher plasmatic levels of PL-C17:0. Elevated abundances of the genus *Fusicatenibacter* and the species *F. saccharivorans* have been associated with cardiovascular risk factors (ApoB, TG, and TC) in adults, where these associations remained significant after adjustment for age, sex, BMI, and alcohol intake [63]. Similarly, we observed that in pediatric T2DM and MetS, *Fusicatenibacter* significance persisted after adjustment for BMI percentile and metformin treatment and positively correlated with altered glucose homeostasis parameters (insulin and HOMA-IR).

As described in the MetS group, the CE-omega-6/omega-3 ratio was significantly higher than those in the T2DM and healthy groups and was associated with a microbiota pattern. High dietary intake of omega-6 has been associated with changes in gut microbiota composition. A previous preclinical study demonstrated that an excess of omega-6 FA from the diet, which resulted in an elevated omega-6/omega-3 ratio, promoted an increase in lipopolysaccharide-producing bacteria (gram-negative), while lipopolysaccharide-alleviating bacteria decreased [64]. In the MetS group, we observed significant and positive correlations between higher levels of omega-6 FAs and the gram-negative bacterial genus *Haemophilus* (within the family *Pasteurellaceae*), and the gram-positive bacteria from the families *Lachnospiraceae* (*Enterocloster* and *Roseburia*) and *Oscillospiraceae* (*Agathobaculum*, *Dysosmobacter*, and *Ventrimonas*) (Fig. 6F). Most of these genera were also positively correlated with obesity parameters and insulin resistance. In adults, *Roseburia inulinivorans* significantly and positively correlated with higher energy and protein intake and showed a trend toward a positive correlation with obesity markers [65]. In the present pediatric cohort, *Agathobaculum*, *Dysosmobacter*, and *Ventrimonas* persisted significantly higher in MetS after adjustment for Tanner stage, sex, BMI percentile, and metformin treatment. Salazar-Jaramillo *et al.* [66] reported a decrease in *Clostridia* (within the *Oscillospiraceae* family), which negatively correlated with BMI.

Microbiota patterns in the MetS group also featured a decrease in the genus *Hungatella*, a *Clostridiaceae* member (phylum Bacillota). In agreement, a previous study in children with obesity also showed a lower abundance of *Hungatella* [67]. On the other hand, the *Lactobacillaceae* family (phylum Bacillota) increased in T2DM in reference to that in the healthy group. Accordingly, previous studies associated an elevated abundance of *Lactobacillaceae* with obesity in adults [68]. However, strains within the *Lactobacillaceae* family have been linked with beneficial effects when used as probiotics, reducing BMI and improving inflammation and insulin resistance [69].

The most abundant genus and species in the pediatric T2DM and MetS subjects were *Prevotella* and *P. copri* (phylum Bacteroidota). In a previous study in adults, *Prevotella_9 copri* was positively correlated with BMI and visceral adiposity, with a trend toward a positive association with carbohydrate and PUFA intake [65]. Also, in this work, both the T2DM and MetS groups had decreased abundances of the family *Marinifilaceae* (phylum Bacteroidota). On the other hand, previously, Carrizalez-Sánchez *et al.* [32] reported an inverse association between the *Odoribacteraceae* and *Bacteroidaceae* families (Bacteroidota phylum) and T2DM and MetS in the present cohort. Accordingly, we identified significantly decreased abundances at the genus (*Odoribacter*) and species (*O. splanchnicus*) levels within the *Odoribacteraceae* family in both groups, T2DM and MetS, and at the genus level (*Bacteroides*) within the *Bacteroidaceae* family in MetS.

An interesting new finding from the present study was the observation of higher abundances of *Butyricimonas* and *Butyricimonas virosa*, also from the *Odoribacteraceae* family, in the MetS children and adolescents. Conversely, a previous study reported a lower abundance of the genus *Butyricimonas* in children with obesity, but in accordance with our results, the family *Marinifilaceae* and *Odoribacter* also decreased [67]. In adults with environmental sensitivity, the family *Marinifilaceae* and the genus *Butyricimonas* were directly linked with C-protein reactive, an inflammatory biomarker [70]. In the present study, the associations of the family *Marinifilaceae* and the genus *Odoribacter* with T2DM and MetS were attenuated after adjustment for BMI percentile and metformin treatment. In contrast, the associations of *Bacteroides* and *Butyricimonas* with MetS remained significant after adjustment, indicating that they were not explained by obesity or metformin treatment.

Comparisons with published studies suggest that bacterial families and genera within the phyla Bacteroidota and Bacillota had distinct profiles associated with disease status and population age (children versus adults). In addition, dietary intake has been associated with influencing gut microbiota modulation [71]; however, it was not assessed in the present work. Therefore, further studies are needed to better characterize microbiota–host interactions across specific populations and disease conditions and to evaluate the contribution of dietary patterns to gut microbiota composition.

## Conclusions

In the present study, children and adolescents with T2DM and MetS featured differential patterns of esterified FAs in plasma lipidic fractions (PLs and CEs). In reference to healthy subjects, the T2DM and MetS groups had higher plasma concentrations of total and individual saturated molecules in PLs (C17:0) and in CEs (C10:0, C12:0, and C24:0) and various omega-6 FAs in PLs (C20:3n-6 and C22:5n-6) and in CEs (C18:2n-6, C20:3n-6, and C20:4n-6). The characteristic plasma FA profiles of pediatric T2DM and MetS were significantly associated with obesity-derived comorbidities after adjustment for Tanner stage and sex. Particularly, PL-C17:0 remained significantly associated with T2DM and MetS after adjustment for obesity-related parameters, HOMA-IR, or metformin medication. These increased FAs were correlated with obesity, abdominal adiposity, dyslipidemia, and insulin resistance. The quantification of esterified FAs enabled a deeper understanding and validation of previous untargeted lipidomic results that suggested altered FA profiles in complex lipids in T2DM and MetS pediatric subjects.

The present study is limited to the observation of FA concentrations released from both plasma fractions (PLs and CEs), which reflect a balance that can be influenced by dietary intake, endogenous metabolism, and, potentially, FAs produced by the gut microbial community. Due to a limited scope, the individual contributions of endogenous and exogenous dietary sources to plasma FA levels cannot be determined. However, based on the observed concentrations of essential PUFAs in the pediatric T2DM and MetS conditions, which included higher CE-LA (C18:2n-6), lower CE-ALA (C18:3n-3), and higher omega-6/omega-3 FA ratios in MetS subjects, a diet rich in omega-6 FAs and low in omega-3 FAs is consistent with the observed lipid profiles.

The present work also contributed to advancing current knowledge of the role of gut microbiota in T2DM and MetS in children and adolescents, a population for which evidence remains scarce. Bacteria from the families *Marinifilaceae* and *Odoribacteraceae* (Bacteroidota phylum) and *Lachnospiraceae* and *Oscillospiraceae* (Bacillota phylum) appeared as distinctly relevant in the gut microbiota dynamics of T2DM and MetS in pediatrics. Gut microbial genera associated with T2DM and MetS after Tanner, sex, and BMI percentile adjustment were *Agathobacter*, *Dorea, Fusicatenibacter*, and *Gemmiger*, with higher abundances than those in healthy controls, and the genus *Faecalimonas* at lower abundances. Further work with the pediatric population affected by T2DM and MetS is needed to continue exploring the observed associations between plasma FA patterns, such as PL-C17:0, and their potential connections with endogenous metabolism, exogenous sources, and gut microbiota.

## Data Availability

The data used in the current study are available from the corresponding authors on reasonable request. The sequencing datasets used in this study are publicly available in the NCBI Sequence Read Archive (SRA) under BioProject PRJNA819279.
