# Supplementary material for: Higher Plasma Saturated and Omega-6 Fatty Acids Associated with Cholesterol Homeostasis and Gut Microbiota in Pediatric Type 2 Diabetes and Metabolic Syndrome
Source: Comput Struct Biotechnol J. 2026 Aug 10;35(1):0194. doi: 10.34133/csbj.0194 (PMC13454464; doi:10.34133/csbj.0194)
Supplement: Supplementary file 1 [file csbj.0194.f1.zip › R1_Supplementary file 1.docx]

**Supplementary Material**

**Fig. S1.** Principal component analysis of significantly different FAs esterified to plasma (**a**) phospholipids (PLs), (**c**) cholesterol esters (CEs), and (**c**) PLs + CEs, in children and adolescents with type 2 diabetes mellitus (T2DM) and metabolic syndrome (MetS), and a healthy group (Kruskal-Wallis´s and Dunn test, FDR *P* < 0.05).

**Fig. S2.** Spearman correlation between fatty acids (FAs) esterified to plasma phospholipids (PLs) and cholesterol esters (CEs). Dot size and color intensity represent the coefficient (red = positive, blue = negative). Asterisks indicate significant correlations: *P* < 0.05 (*), *P* < 0.01 (**), *P* < 0.001 (***).

**Fig. S3**. Alpha diversity indices at the genus level in children and adolescents with type 2 diabetes mellitus (T2DM) and metabolic syndrome (MetS) and a healthy group.

**Fig. S4**. Relative microbial composition at the phylum level in children and adolescents with type 2 diabetes mellitus (T2DM) and metabolic syndrome (MetS), and a healthy group.

**Fig. S5**. Relative microbial composition at the family level in children and adolescents with type 2 diabetes mellitus (T2DM) and metabolic syndrome (MetS), and a healthy group.

**Fig. S6**. Relative microbial composition at the genus level in children and adolescents with type 2 diabetes mellitus (T2DM) and metabolic syndrome (MetS), and a healthy group.

**Fig. S7**. Relative microbial composition at the species level in children and adolescents with type 2 diabetes mellitus (T2DM) and metabolic syndrome (MetS), and a healthy group.
